# Influence of genetic factors on long-term treatment related neurocognitive complications, and on anxiety and depression in survivors of childhood acute lymphoblastic leukemia: The Petale study

**DOI:** 10.1371/journal.pone.0217314

**Published:** 2019-06-10

**Authors:** Kateryna Petrykey, Sarah Lippé, Philippe Robaey, Serge Sultan, Julie Laniel, Simon Drouin, Laurence Bertout, Patrick Beaulieu, Pascal St-Onge, Aubrée Boulet-Craig, Aziz Rezgui, Yutaka Yasui, Yadav Sapkota, Kevin R. Krull, Melissa M. Hudson, Caroline Laverdière, Daniel Sinnett, Maja Krajinovic

**Affiliations:** 1 Sainte-Justine University Health Center (SJUHC), Montreal, Quebec, Canada; 2 Department of Pharmacology and Physiology, Université de Montréal, Montreal, Quebec, Canada; 3 Department of Psychology, Université de Montréal, Montreal, Quebec, Canada; 4 Children’s Hospital of Eastern Ontario, Ottawa, Ontario, Canada; 5 Department of Psychiatry, Université de Montréal, Montreal, Quebec, Canada; 6 Department of Psychiatry, University of Ottawa, Ottawa, Ontario, Canada; 7 Epidemiology and Cancer Control Department, St. Jude Children’s Research Hospital, Memphis, TN, United States of America; 8 Oncology Department, St. Jude Children’s Research Hospital, Memphis, TN, United States of America; 9 Department of Pediatrics, Université de Montréal, Montreal, Quebec, Canada; Unicamillus, Saint Camillus International University of Health Sciences, ITALY

## Abstract

**Background:**

A substantial number of survivors of childhood acute lymphoblastic leukemia suffer from treatment-related late adverse effects including neurocognitive impairment. While multiple studies have described neurocognitive outcomes in childhood acute lymphoblastic leukemia (ALL) survivors, relatively few have investigated their association with individual genetic constitution.

**Methods:**

To further address this issue, genetic variants located in 99 genes relevant to the effects of anticancer drugs and in 360 genes implicated in nervous system function and predicted to affect protein function, were pooled from whole exome sequencing data of childhood ALL survivors (PETALE cohort) and analyzed for an association with neurocognitive complications, as well as with anxiety and depression. Variants that sustained correction for multiple testing were genotyped in entire cohort (n = 236) and analyzed with same outcomes.

**Results:**

Common variants in *MTR*, *PPARA*, *ABCC3*, *CALML5*, *CACNB2* and *PCDHB10* genes were associated with deficits in neurocognitive tests performance, whereas a variant in *SLCO1B1* and *EPHA5* genes was associated with anxiety and depression. Majority of associations were modulated by intensity of treatment. Associated variants were further analyzed in an independent SJLIFE cohort of 545 ALL survivors. Two variants, *rs1805087* in methionine synthase, *MTR* and *rs58225473* in voltage-dependent calcium channel protein encoding gene, *CACNB2* are of particular interest, since associations of borderline significance were found in replication cohort and remain significant in combined discovery and replication groups (OR = 1.5, 95% CI, 1–2.3; p = 0.04 and; OR = 3.7, 95% CI, 1.25–11; p = 0.01, respectively). Variant *rs4149056* in *SLCO1B1* gene also deserves further attention since previously shown to affect methotrexate clearance and short-term toxicity in ALL patients.

**Conclusions:**

Current findings can help understanding of the influence of genetic component on long-term neurocognitive impairment. Further studies are needed to confirm whether identified variants may be useful in identifying survivors at increased risk of these complications.

## Introduction

Acute lymphoblastic leukemia (ALL) is the most frequent childhood cancer [1] accounting for approximately 25% of all cases [2]. The five-year survival rate of childhood ALL is currently greater than 85% due to the optimization of multi-agent risk-adapted treatment strategies [2, 3]. However, the exposure to specific chemotherapeutic agents and/or cranial radiation therapy during a susceptible period of child development results in late-adverse effects (LAEs) [4–6] including neurocognitive impairments [2]. Clinically significant deficits among ALL survivors are most commonly found in attention [7–12], working memory [13], processing speed [9, 14, 15] and executive functions, such as verbal fluency and cognitive flexibility [16]. Neurocognitive impairment in childhood ALL survivors persist for many years after treatment [17, 18]. Large survey studies like the Childhood Cancer Survivors Study (CCSS) as well as other studies conducted in childhood ALL survivors [7, 9, 19] have demonstrated higher risk of depression, anxiety, behavioural difficulties, distress, as well as post-traumatic symptoms compared to siblings [20–25]. Longitudinal follow-up in long-term survivors have indicated that frequency of distress evolves over time, with more than 10% of survivors experiencing significant increase in depression as well as in anxiety [26].

The childhood ALL survivor population is increasing in size and lifespan, and this specific population needs an effective evaluation and targeted interventions [27]. Thus, better understanding of LAEs and factors contributing to their development is important to guide survivorship health surveillance and strategies to prevent or remediate treatment-related toxicities [2, 3]. Here we assessed the role of genetic factors in neurocognitive impairments as well as in anxiety and depression by interrogating the relationship between above mentioned complications and genotypic profiling of 459 candidate genes obtained through whole exome sequencing (WES) of childhood ALL survivors.

## Study population and methods

### Discovery cohort

The discovery cohort included 236 patients diagnosed and treated for childhood ALL according to Dana Farber Cancer Institute (DFCI) ALL 87–01 to 05–01 protocols at Sainte-Justine University Health Center (SJUHC), Montreal, (Quebec), Canada. The participants were recruited during 2013–2015 in the context of the PETALE study, a multidisciplinary research project with the goal to identify and comprehensively characterize associated predictive biomarkers of long-term treatment related complications in childhood ALL survivors [6]. Eligible participants were younger than 19 years old at diagnosis, at least 5 years after diagnosis of ALL and older than 12 years at evaluation, without history of relapse or refractory ALL or neurological condition or Down syndrome and had not received a hematopoietic stem cell transplant. The time from end of treatment to evaluation ranged from 5–26 year with a median of 13 years. The patients were classified to standard (SR) and high risk (HR) groups based on prognostic factors, including age, white blood cell count, immunophenotype, and central nervous system (CNS) status at diagnosis [28, 29]. The frequency of patients assigned to SR and HR groups during the treatment was 46.6% and 53.4%, respectively. They were almost exclusively of reported French Canadian descent (>95%). The HapMap genotype reference data [30, 31] was used for Principal component analysis (PCA) [30] to test and confirm predominant European ancestry (**S1 Fig**).

### Replication cohort

The replication cohort consisted of 545 ALL survivors (274 males and 271 females) of European ancestry (based on genotype data) enrolled in the St. Jude Lifetime Cohort (SJLIFE) study and were evaluated using the same testing procedures as in PETALE cohort. Participants were younger than 19 years old at diagnosis and younger than 40 years old at SJLIFE evaluation, with no diagnosis of neurologic condition or Down syndrome, no history of relapse and had not received a hematopoietic stem cell transplant. The time from primary cancer diagnosis to the most recent date of neurocognitive evaluation ranged from 10.95–45.60 years with a median of 26.84 years. The risk group assignment during the treatment (SR and HR groups) was not available for this cohort.

### Neuropsychological evaluation

A neurocognitive evaluation was performed using standardized testing procedures. It included three indices from two cognitive measures that reflect common impairments among childhood ALL survivors and are also good predictors of general neuropsychological outcomes [32]: Trail Making Test–Condition 4—Letter-Number Sequencing score and Verbal Fluency–Condition 1 –Letter fluency score from the Delis-Kaplan Executive Function System (D-KEFS) [33]; and Digit Span from the Wechsler Adult Intelligence Scale-Fourth Edition (WAIS-IV) [34] total score. Trail Making Test (D-KEFS) score is a measure that reflects processing speed, psychomotor speed, and cognitive flexibility [35]. Verbal Fluency (D-KEFS) score is a measure of phonological fluency in verbal modality [36]. Digit span (WAIS-IV) total score is a measure of verbal working memory [37]. All raw scores were converted to age-adjusted scaled scores based on population means [38]. The neurocognitive outcomes were transformed into dichotomous variables and studied accordingly. For each of these variables, scores lower than one and a half standard deviations below the mean of the normative dataset were indicative of impairment [39], all other scores were considered non-impaired.

### Emotional distress: Anxiety and depression

Participants were classified having emotional distress if they demonstrated elevated symptoms according to two measures referenced to age-specific norms. This was done in line with published recommendations [40, 41] and previous use of the instruments [21, 22, 26]. For younger participants (<19 years), we used anxiety and depression modules of the Beck Youth Inventory (BYI), a self-report instrument to document psychological status in children from 7 to 18 years old [41]. For older participants (≥19 years) we used the Brief Symptom Inventory-18 (BSI-18 anxiety and depression score), an 18-item self-report questionnaire, assessing psychological distress in adults [40], previously also used in cohorts of young and older adult survivors of childhood cancer [42, 43]. Internal consistency coefficients measured by Cronbach’s alphas were all satisfactory, >.80 [44]. Age-adjusted scores one standard deviation above the population mean were considered as impaired.

### Sequencing and quality control

Whole-exome sequencing (WES) was performed on germline DNA, extracted from peripheral blood samples from a subset of 191 participants of PETALE cohort, using standard protocols as described previously [6]. Whole exomes were captured in solution with Agilent’s SureSelect Human All Exon 50Mb kits and sequenced on either Life Technologies SOLiD System 4.0 (mean coverage = 40X) or Illumina HiSeq 2500 platform (mean coverage = 113.1X) at SJUHC integrated clinical genomic centre in pediatrics. Reads were aligned to the hg19 reference genome using SOLiD LifeScope software [45] for the SOLiD samples and BWA-MEM [46] for the samples sequenced on the Illumina system. PICARD [47, 48] was used to mark PCR duplicates and collect sequencing quality control metrics. Variant calling was performed using the Haplotype Caller and quality score recalibration was performed using Variant Recalibrator, both implemented in the Genome Analysis Tool Kit (GATK) [48]. Variants were selected based on the variant quality score (VQSR = PASS) and minimum depth of coverage (DP > = 10). The final germline variants were annotated by ANNOVAR [49] and the predicted functional impact of missense, nonsense and splicing common and rare variants was assessed in silico using Sift (<0.1) and PolyPhen2 (≥0.85) filters [50, 51]. Variants were defined as rare (minor allele frequency, MAF<5%) and common (MAF≥5%) according to the reported frequency for European populations in the 1000 Genomes [52] and ESP6500 datasets [53]. These variants were considered as potentially damaging and were used for analyses. Variants exceeding missing rate of 20%, with minor allele count<2 and not in Hardy-Weinberg Equilibrium (*P*<0.001) were excluded.

### Association analyses

Two sets of candidate genes were selected—the first consisted of 99 genes implicated in the metabolism of methotrexate (MTX) and corticosteroids (CS) which are known to impact neurocognitive outcomes [54, 55] and the second consisted of 360 genes implicated in nervous system function, selected using the KEGG PATHWAY Database [56]. A total of 76 common variants (27 in MTX/CS pathway and 49 in nervous system function) and 1337 rare variants that satisfied all above filtering criteria were identified as functionally predicted and were used in association analyses. The analyses between common genetic variants and neurocognitive outcomes as well as with anxiety/depression were performed by the allelic chi-square or Fisher’s exact test implemented in PLINK v.1.07 [57, 58]. Analyses were performed in 191 sequenced patients and stratified by sex, risk groups with different treatment intensity, and treatment with chemotherapy alone or chemotherapy and cranial radiation because these factors have an established role in modulating neurocognitive outcomes [4, 59]. The Benjamini-Hochberg procedure for false discovery rate (FDR) [60, 61] was used to adjust for multiple testing with a cut-off value of < 5% considered statistically significant. Selective genotyping of top-ranking common SNPs (based additionally on Bonferroni p-value corrected for the number of variants tested, p<0.001 and p<0.0019 for the neural and MTX/CS pathways, respectively) was carried out on the Sequenom platform at the McGill University and Génome Québec Innovation Centre, Montreal, (Quebec), Canada, to confirm the results and extend the analysis to entire cohort (n = 236) including one hundred ninety-one patients analysed above (**S1 Table**). Associations of genotyped variants with the outcomes were assessed using chi-square or Fisher exact test in SPSS v.24.0.0.0 and appropriate genetic models, which were presented relative to the minor allele. Genotype-outcome association was represented as an odds ratio (OR) with a 95% confidence interval (CI). For the variants of MTX pathway for which the association showed similar trend in validation cohort, the modulation of the effect by cumulative drug dose was also analyzed. For that, cumulative drug dose was dichotomized to above and below the median and the association was analyzed in each subgroup. Additionally, logistic regression model was used in which main effect (genotype and drug dose) and interaction term were added. The detailed list of the studied polymorphisms **(DOI: 10.6084/m9.figshare.8051573)**, as well as the summary statistics for all polymorphisms analyzed from the sequencing data beyond those already presented in the regular and supplemental tables **(DOI: 10.6084/m9.figshare.8051825)** are provided.

For rare variants associations, we used the SKAT-O test (Optimal Sequence Kernel Association Test) [62, 63] implemented in SKAT package v.1.3.2.1 [64] with FDR < 5% considered statistically significant. Collapsing approach that combines several rare variants into a single variable [65, 66], with iterative exclusion of each single variant, was additionally performed to allow weighting variant contributions to association signals. These analyses were performed as exploratory and associated variants were not further analyzed by genotyping.

### Replication analysis

Genotype data for selected variants were obtained from a larger effort to sequence whole-genomes of over three thousand long-term survivors participating in the SJLIFE cohort. For this replication analysis, we restricted inclusion to 545 ALL survivors of European ancestry. Associations of selected variants with respective neurocognitive outcomes were examined using chi-square or Fisher’s exact tests, as appropriate, implemented in PLINK 1.9 [57, 58].

## Results

### Neurocognitive and emotional disturbances

The median age of ALL survivors at the time of evaluation was 21 years, with almost equal sex distribution, their demographics and clinical characteristics are presented in **Table 1**. The most prevalent deficit in neurocognitive test performance was noted for digit span (19.5%) followed by verbal fluency (18.6%) and trail making test (9.3%). Moderate-severe anxiety was noted in 10.1% survivors, whereas 11.5% of survivors were affected by moderate-severe depression, which was comparable to published normative groups on anxiety and depression [40–42].

**Table 1:** Patient demographics and clinical characteristics (N = 236).

		N	%
**Sex**			
	Male	115	48.7
	Female	121	51.3
**Neuropsychological outcomes (affected patients)**			
	Trial making test*	22	9.3
	Verbal fluency*	44	18.6
	Digit span*	46	19.5
	Moderate-severe anxiety	21	10.1
	Moderate-severe depression	24	11.5
**DFCI protocol**			
	87–01	18	7.6
	91–01	48	20.3
	95–01	71	30.1
	00–01	75	31.8
	05–01	24	10.2
**Prognostic risk group**			
	Standard risk (SR)	110	46.6
	High risk (HR)	126	53.4
**Cranial radiation therapy**			
	Yes**	131	55.5
	No	105	44.5
	**Cumulative doses, mg/m2—median (range)**		
	Parenteral/PO MTX (853.6–12750.5)	6576.5	
	IT MTX (0–279)	150.4	
	IV/PO corticosteroids*** (4425.7–24930.1)	8826.6	
	IT corticosteroids*** (2.05–87.19)	31.3	
**Age at diagnosis—median (range)**			
	Male (1–18)	5.0	
	Female (0–18)	4.0	
**Age at follow—up median (range)**			
	Male (12–36)	21.0	
	Female (12–38)	21.0	

DFCI, Dana-Farber Cancer Institute; IV, intravenous; PO, per os; IT, intrathecal; MTX, methotrexate

*Score at least 1.5 standard deviation below the norm was considered as impaired in all neuropsychological tests

** Median (range), 12 Gy (12-18Gy)

*** Cumulative corticosteroid doses are calculated as prednisone equivalents

### Common variants

Among common variants implicated in nervous system function obtained from WES data, significant associations were detected for four of them (*CALML5*, *CACNB2*, *PCDHB10* and *EPHA5)* either in all survivors or following stratification according to sex, risk groups or CRT (**S2 Table**). These variants were further analyzed by genotyping in the entire PETALE cohort and the association was confirmed for all of them (**Table 2**). The analyses were performed for the same subgroups for which association was noted for WES data, and additionally in all participants. The neurocognitive deficit related to digit span task was associated in an additive manner with the minor allele of *rs58225473* in *CACNB2* gene either in all patients (p = 0.02), or those who received chemotherapy only (p = 0.004). Homozygotes for the minor C allele of *CALML5 rs10904516* were at higher risk of having deficit in verbal fluency score, whereas the neurocognitive deficit related to trail making test was associated with the minor allele of *rs2907323* in *PCDHB10* gene, both potentiated in HR participants (p = 0.03 and p = 0.01 respectively). The carriers of the minor C allele of *EPHA5 rs33932471* were at higher risk of both moderate-severe anxiety and depression, with the strongest effects seen in females (p = 0.02 and p = 0.003, respectively).

**Table 2:** Frequency of associated genotypes in patients with and without neurocognitive or emotional distress, genes of relevance for nervous system function, PETALE cohort (N = 236).

Outcome	Genotype	Case*, N (%)	Control*, N (%)	Model	Case*, N (%)	Control*, N (%)	P**	OR (95%-CI)
**Digit span**	***CACNB2 rs58225473***							
	**All patients**							
	**TT**	25 (58.1)	128 (71.5)	**TT**	25 (58.1)	128 (71.5)	0.02^a^	2.0 (1.1–3.9)
	**TG**	16 (37.2)	51 (28.5)	**TG**	16 (37.2)	51 (28.5)		
	**GG**	2 (4.7)	0 (0.0)	**GG**	2 (4.7)	0 (0.0)		
	Chemotherapy only***							
	**TT**	4 (40.0)	63 (70.8)	**TT**	4 (40.0)	63 (70.8)	0.004^a^	5.0 (1.5–16.4)
	**TG**	4 (40.0)	26 (29.2)	**TG**	4 (40.0)	26 (29.2)		
	**GG**	2 (20.0)	0 (0.0)	**GG**	2 (20.0)	0 (0.0)		
	***PCDHB10 rs2907323***							
**Trial making test**	**All patients**							
	**GG**	11 (55.0)	155 (73.1)	**GG**	11 (55.0)	155 (73.1)		
	**GC**	8 (40.0)	57 (26.9)	**GC**	8 (40.0)	57 (26.9)	0.02^a^	2.5
	**CC**	1 (5.0)	0 (0.0)	**CC**	1 (5.0)	0 (0.0)		(1.1–6.2)
	**High risk**							
	**GG**	6 (46.2)	85 (76.6)	**GG**	6 (46.2)	85 (76.6)		
	**GC**	6 (46.2)	26 (23.4)	**GC**	6 (46.2)	26 (23.4)	0.01^a^	4.3
	**CC**	1 (7.7)	0 (0.0)	**CC**	1 (7.7)	0 (0.0)		(1.4–12.7)
**Verbal fluency**	***CALML5 rs10904516***							
	**All patients**																
	**TT**	18 (40.9)	93 (48.7)	**TT+TC**	37 (84.1)	178 (93.2)	0.05^r^	2.6 (1.0–6.9)
	**TC**	19 (43.2)	85 (44.5)					
	**CC**	7 (15.9)	13 (6.8)	**CC**	7 (15.9)	13 (6.8)		
	**High risk**							
	**TT**	8 (34.8)	52 (50.5)	**TT+TC**	18 (78.3)	96 (93.2)	0.03^r^	3.8 (1.1–13.3)
	**TC**	10 (43.5)	44 (42.7)					
	**CC**	5 (21.7)	7 (6.8)	**CC**	5 (21.7)	7 (6.8)		
**Moderate-severe anxiety**	***EPHA5 rs33932471***							
	**Females**							
	**AA**	5 (55.6)	82 (89.1)	**AA**	5 (55.6)	82 (89.1)	0.02^d^	6.6 (1.5–28.5)
	**AC**	4 (44.4)	8 (8.7)	**AC+CC**	4 (44.4)	10 (10.9)		
	**CC**	0 (0.0)	2 (2.2)					
**Moderate-severe depression**	***EPHA5 rs33932471***							
	**All patients**							
	**AA**	15 (78.9)	151 (88.8)	**AA**	15 (71.4)	151 (88.8)	0.03^d^	3.2 (1.1–9.2)
	**AC**	6 (28.6)	17 (10.0)	**AC+CC**	6 (28.6)	19 (11.2)		
	**CC**	0 (0.0)	2 (1.2)					
	**Females**							
	**AA**	7 (58.3)	80 (89.9)	**AA**	7 (58.3)	80 (89.9)	0.003^d^	6.3 (1.7–24.2)
	**AC**	5 (41.7)	7 (7.9)	**AC+CC**	5 (41.7)	9 (10.1)		
	**CC**	0 (0.0)	2 (2.2)					

*CACNB2*: Calcium Voltage-Gated Channel Auxiliary Subunit Beta2; *CALML5*: Calmodulin Like 5, *EPHA5*: EPH Receptor A5, Brain-Specific Kinase; OR, odds ratio; CI, confidence interval.

*Participants with and without indicated complications are defined as cases and controls, respectively.

**P values are calculated by chi-square or Fisher exact test, as appropriate. The most representative genetic model used is indicated (a: Additive; d: Dominant, r: Recessive).

***Chemotherapy without cranial radiation therapy.

Among common variants implicated in MTX/CS pathway obtained from WES data, the significant associations were detected for 6 of them (*MTR*, *PPARA*, *ABCC3*, *SHMT1* and *SLCO1B1*, (**S3 Table**). The variants in *MTR*, *PPARA*, *ABCC3* and *SLCO1B1* genes were further analyzed by genotyping in entire PETALE cohort and the association was confirmed for all of them (**Table 3**). The association between deficit in verbal fluency score and GG genotype of *MTR rs1805087* was seen for all survivors (p = 0.01) and male participants (p = 0.002). Deficits in verbal fluency performance were also associated with GG genotype of *ABCC3 rs12604031* among HR patients (p = 0.001) as well as with *rs1800206* in *PPARA* gene in low risk groups (p = 0.008). The risk of moderate-severe depression was highest among carriers of the minor G allele of *SLCO1B1 rs4149056* who received chemotherapy only (p = 0.002).

**Table 3:** Frequency of associated genotypes in patients with and without neurocognitive or emotional distress, genes implicated in methotrexate and corticosteroids pathways, PETALE cohort (N = 236).

Outcome	Genotype	Case*, N (%)	Control*, N (%)	Model	Case*, N (%)	Control*, N (%)	P**	OR (95%-CI)
**Verbal fluency**	***MTR rs1805087***							
	**All patients**							
	**AA**	25 (61.0)	120 (66.7)	**AA+AG**	37 (90.2)	178 (98.9)	0.01^r^	9.6 (1.7–54.5)
	**AG**	12 (29.2)	58 (32.2)					
	**GG**	4 (9.8)	2 (1.1)	**GG**	4 (9.8)	2 (1.1)		
	**Males**							
	**AA**	12 (52.2)	57 (66.3)	**AA+AG**	19 (82.6)	86 (100.0)	0.002^r^	
	**AG**	7 (30.4)	29 (33.7)					
	**GG**	4 (17.4)	0 (0.0)	**GG**	4 (17.4)	0 (0.0)		
	***PPARA rs1800206***							
	**Standard risk**							
	**CC**	13 (72.7)	76 (92.7)	**CC**	13 (72.7)	76 (92.7)	0.008^a^	4.6 (1.5–14.5)
	**CG**	3 (16.7)	6 (7.3)	**CG**	3 (16.7)	6 (7.3)		
	**GG**	2 (11.1)	0 (0.0)	**GG**	2 (11.1)	0 (0.0)		
	**Chemotherapy only**							
	**CC**	12 (75.0)	77 (92.8)	**CC**	12 (75.0)	77 (92.8)	0.02^a^	4.3 (1.3–13.5)
	**CG**	2 (12.5)	6 (7.2)	**CG**	2 (12.5)	6 (7.2)		
	**GG**	2 (12.5)	0 (0.0)	**GG**	2 (12.5)	0 (0.0)		
	***ABCC3 rs12604031***							
	**High risk**							
	**AA**	3 (13.0)	33 (33.3)	**AA+AG**	14 (60.9)	89 (89.9)	0.001^r^	5.7 (2.0–16.5)
	**AG**	11 (47.8)	56 (56.6)					
	**GG**	9 (39.1)	10 (10.1)	**GG**	9 (39.1)	10 (10.1)		
**Moderate-severe depression**	***SLCO1B1 rs4149056***							
	**Chemotherapy only**							
	**AA**	1 (14.3)	59 (75.6	**AA**	1 (14.3)	59 (76.5)	0.002^d^	18.6 (2.1–164.7)
	**AG**	5 (71.4)	19 (24.4)	**AG+GG**	6 (85.7)	19 (24.4)		
	**GG**	1 (14.3)	0 (0.0)					

*MTR*: 5-Methyltetrahydrofolate-Homocysteine Methyltransferase, *PPARA*: Peroxisome Proliferator Activated Receptor Alpha, *ABCC3*: ATP Binding Cassette Subfamily C Member 3, *SLCO1B1*: Solute Carrier Organic Anion Transporter Family Member 1B1.

*Participants with and without indicated complications are defined as cases and controls, respectively.

**P values are calculated by chi-square or Fisher exact test, as appropriate. The most representative genetic model used is indicated (a: Additive; d: Dominant, r: Recessive).

All variants found significantly associated with tested outcome (except those initially confined to risk subgroup such as those in *ABCC3* and *PCDHB10* genes) were further analyzed for an association with respective outcomes in an independent cohort of ALL survivors (SJLIFE cohort) **(Tables 4 and 5)**. Two associations were noticeable. The association of borderline significance between deficit in verbal fluency score and the minor allele of *MTR rs1805087* was seen in all survivors (OR = 1.7; 95% CI, 1.0–2.8; p = 0.05). The association between deficit in digit span score and GG genotype of *CACNB2 rs58225473* showed similar trend as in PETALE cohort for all participants (OR = 3.7, 95% CI, 1.0–13.9), as well as for patients who received chemotherapy only (OR = 3.8, 95% CI, 0.9–16.5), however they did not reach significance (p = 0.08 and 0.09, respectively). The associations for *rs58225473* and *rs1805087* variants in *CACNB2* and *MTR* genes were significant for combined PETALE and SJLIFE cohorts (**S4 Table**). The association between deficit in digit span score and GG genotype of *CACNB2 rs58225473* was significant for all participants (OR = 3.7; 95% CI, 1.25–11; p = 0.01) and for patients who received chemotherapy only (OR = 7.2, 95% CI, 2.1–25; p = 0.0004). The association between deficit in verbal fluency score and the minor allele of MTR rs1805087 was seen in all survivors (OR = 1.5; 95% CI, 1–2.3; p = 0.04) and in male participants (OR = 1.8; 95% CI, 1–3.1; p = 0.04).

**Table 4:** Frequency of genotypes in patients with and without neurocognitive or emotional distress, genes of relevance for nervous system function, replication cohort of SJLIFE (N = 545).

Outcome	Genotype	Case*N (%)	Control* N (%)	Model	Case* N (%)	Control* N (%)	P**	OR (95%-CI)
**Digit span**	***CACNB2 rs58225473***							
	**All patients**							
	**TT**	23 (71.8)	279 (69.1)	**TT+TG**	29 (90.6)	393 (97.3)	0.08^r^	3.7 (1.0–13.9)
	**TG**	6 (18.8)	114 (28.2)					
	**GG**	3 (9.4)	11 (2.7)	**GG**	3 (9.4)	11 (2.7)		
	**Chemotherapy only**							
	**TT**	16 (66.7)	112 (67.1)	**TT+TG**	21 (87.5)	161 (96.4)	0.09^r^	3.8 (0.9–16.5)
	**TG**	5 (20.8)	49 (29.3)					
	**GG**	3 (12.5)	6 (3.6)	**GG**	3 (12.5)	6 (3.6)		
**Verbal fluency**	***CALML5 rs10904516***							
	**All patients**							
	**TT**	32 (45.7)	180 (40.0)	**TT+TC**	62 (88.6)	387 (86.0)	0.6^r^	0.8 (0.4–1.7)
	**TC**	30 (42.9)	207 (46.0)					
	**CC**	8 (11.4)	63 (14.0)	**CC**	8 (11.4)	63 (14.0)		
**Moderate-severe anxiety**	***EPHA5 rs33932471***							
	**Females**							
	**AA**	27 (84.4)	205 (87.2)	**AA**	27 (84.4)	205 (87.2)	0.7^d^	1.3 (0.5–3.5)
	**AC**	5 (15.6)	27 (11.5)	**AC+CC**	5 (15.6)	30 (12.8)		
	**CC**	0 (0.0)	3 (1.3)					
**Moderate-severe depression**	***EPHA5 rs33932471***							
	**All patients**							
	**AA**	58 (79.5)	399 (86.0)	**AA**	58 (79.5)	399 (86.0)	0.1^d^	1.6 (0.8–3.0)
	**AC**	15 (20.5)	60 (12.9)	**AC+CC**	15 (20.5)	65 (14.0)		
	**CC**	0 (0.0)	5 (1.1)					
	**Females**							
	**AA**	30 (90.9)	202 (86.3)	**AA**	30 (9.9)	202 (86.3)	0.5^d^	0.6 (0.2–2.2)
	**AC**	3 (9.1)	29 (12.4)	**AC+CC**	3 (9.1)	32 (13.7)		
	**CC**	0 (0.0)	3 (1.3)					

*Participants with and without indicated complications are defined as cases and controls, respectively

**P values are calculated by chi-square or Fisher exact test, as appropriate. The most representative genetic model used is indicated (d: Dominant, r: Recessive).

**Table 5:** Frequency of genotypes in patients with and without neurocognitive or emotional distress, genes implicated in methotrexate and corticosteroids pathways, replication cohort of SJLIFE (N = 545).

Outcome	Genotype	Case* N (%)	Control* N (%)	Model	Case* N (%)	Control* N (%)	P**	OR (95%-CI)
**Verbal fluency**	***MTR rs1805087***							
	**All patients**							
	**AA**	42 (59.2)	323 (70.7)	**AG+GG**	29 (40.8)	134 (29.3)	0.05^d^	1.7(1.0–2.8)
	**AG**	25(35.2)	121 (26.5)					
	**GG**	4 (5.6)	13 (2.8)	**AA**	42 (59.2)	323 (70.7)		
	**Males**							
	**AA**	23 (60.5)	164 (72.2)	**AG+GG**	15 (39.5)	63 (27.8)	0.1^d^	1.7 (0.8–3.5)
	**AG**	13 (34.2)	56 (24.7)					
	**GG**	2 (5.3)	7 (3.1)	**AA**	23 (60.5)	164 (72.2)		
	***PPARA rs1800206***							
	**Chemotherapy only**							
	**CC**	39 (81.2)	155 (85.6)	**CC**	39 (73.8)	155 (85.6)	0.5^d^	1.4 (0.6–3.2)
	**CG**	9 (18.8)	26 (14.4)	**CG+GG**	9 (23.8)	26 (14.4)		
	**GG**	0 (0)	0 (0)					
**Moderate-severe depression**	***SLCO1B1 rs4149056***							
	**Chemotherapy only**							
	**AA**	31 (73.8)	142 (72.4)	**AA**	31 (73.8)	142 (72.4)	0.9^d^	0.9 (0.4–2.0)
	**AG**	10 (23.8)	48 (24.5)	**AG+GG**	11 (26.2)	54 (27.6)		
	**GG**	1 (2.4)	6 (3.1)					

*Participants with and without indicated complications are defined as cases and controls, respectively

**P values are calculated by chi-square or Fisher exact test, as appropriate. The most representative genetic model used is indicated (d: Dominant).

**Fig 1:**
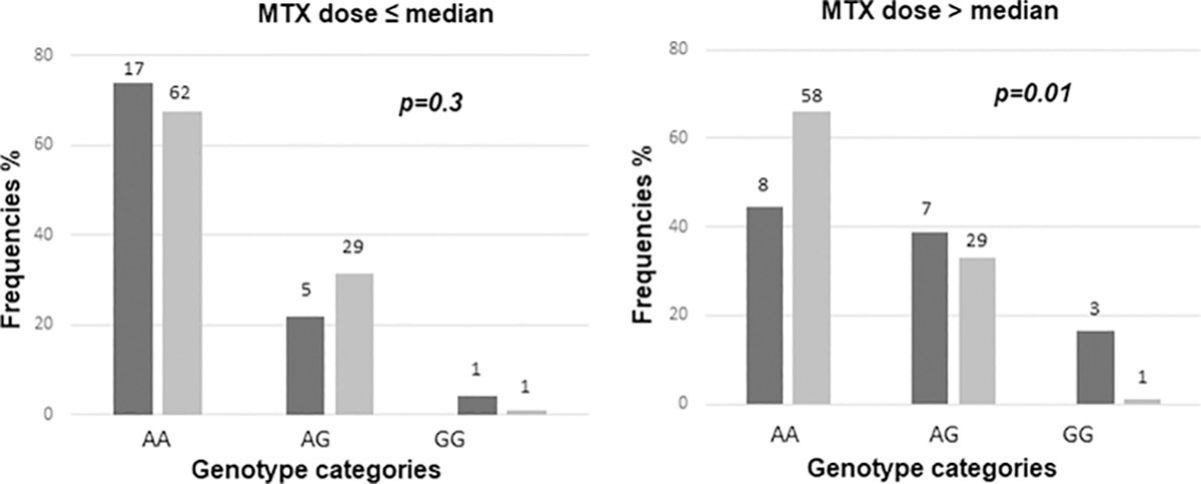
Interaction between MTR rs1805087 and cumulative methotrexate dose. The frequency of each genotype in affected and non-affected group defined by verbal fluency score is presented by black and gray bars, respectively, in the groups that received cumulative MTX below median (left panel) or above median (right panel). The number of individuals represented by each bar and p values are indicted on the plot. OR for interaction is 3.3, 95% CI 0.9–11.5, p = 0.07, as derived from logistic regression model including MTR genotype, cumulative MTX dose and interaction term.

Given that the *MTR* belongs to the MTX pathway, we further explored whether the effect of *rs1805087* was modulated by cumulative MTX doses, for which such data were available in the discovery group (**Table 1**). The relationship with the deficit in verbal fluency score was particularly obvious in patients who received higher overall cumulative doses (**Fig 1**, p = 0.01 for patients with cumulative doses above median vs. p = 0.3, for patients with cumulative doses below median).

### Rare variants

The analysis of functionally predicted rare variants in PETALE cohort led to the detection of an association between the deficit in trail making test score and rare variants enrichment in *SLCO2B1*, *HSPA4* and *GSTT1* genes (p = 0.0002, p = 0.004 and p = 0.003, respectively, **Table 6**). Using the collapsing approach, we explored variant combinations that contributed to the observed association signal, identifying two variants in *GSTT1*, three in *HSPA4* and four in *SLCO2B1* gene. Replication analyses were not performed for these findings because information regarding these variants was not available in the SJLIFE cohort.

**Table 6:** SKAT-O analysis of the rare functional variants, PETALE cohort, WES data, demonstrated for the deficit in trail making test scores (N = 191).

Outcome	Gene	SNPs tested		MAF	P value	FDR	OR^1^ [95% CI]
		position	rs number				
**Trail making test**	***SLCO2B1***	chr11:74875089–74875089		0.009	0.0002	0.004	8.7 [1.3–57.0]
		chr11:74880370–74880370	*rs35199625*	0.008			
		chr11:74899276–74899276	*rs377133671*	0.003			
	***HSPA4***	*chr5:132387979–132387979	***rs61745470***	0.017	0.004	0.027	*7.8 [1.7–36.4]
		chr5:132408967–132408967	*rs61755724*	0.042			
		*chr5:132412511–132412511	* *	0.003			
		*chr5:132437499–132437499	*rs61749631*	0.005			
	***GSTT1***	chr22:24379402–24379402	*rs11550605*	0.003	0.003	0.027	19.7 [1.7–230.6]
		chr22:24381742–24381742		0.006			

SNP: single nucleotide polymorphism; OR: odds ratio; CI: confidence interval; *SLCO2B1*: Solute Carrier Organic Anion Transporter Family Member 2B1; *HSPA4*: Heat Shock Protein Family A (Hsp70) Member 4; *GSTT1*: Glutathione S-Transferase Theta 1. Individual contribution of variant *rs61745470* (noted in bold) was identified with OR = 19.5, 95%CI [2.97–128], p = 0.005.

^**1**^ OR of significant combination, combination include all variants or variants noted with asterisk*.

## Discussion

Functionally predicted germline common variants in *MTR*, *PPARA*, *SLCO1B1*, *ABCC3*, *CALML5*, *CACNB2* and *PCDHB10* genes were found to be significantly associated with deficits in neurocognitive tests performance, whereas a variant in *EPHA5* gene was significantly associated with both anxiety and depression.

### Neurocognitive performance

Among common variants associated with an impairment in neurocognitive function, *rs10904516* in the *MTR* gene, which was associated with a deficit in verbal fluency, seems particularly interesting given similar observation in the SJLIFE cohort. The *MTR* gene encodes a B12 dependent methionine synthase involved in remethylation of homocysteine (Hcy), which is the crucial step in methionine production in all types of cells [67]. Mutations in the *MTR* gene as well as severe deficiency of vitamin B12 could result in elevated concentration of Hcy in plasma and cerebrospinal fluid. Studies have shown that Hcy exerts a neurotoxic action and may participate in the mechanisms of neurodegeneration, such as excitotoxicity, oxidative stress, calcium accumulation, and apoptosis[68–70]. *MTR* gene is involved in the metabolic pathway of MTX. Administration of MTX was associated with acute and subacute neurotoxic effects; these detrimental effects may accumulate over time [69]. The detected common functional polymorphism (*rs1805087*) leading to Asp919Gly amino acid replacement in the *MTR* gene could affect enzymatic activity, thus increasing the level of Hcy [68, 69, 71]. Indeed, we have shown interaction between *MTR rs1805087* and cumulative MTX dose in survivors with the deficit in verbal fluency score. This confirms previous finding: this variant together with polymorphisms of other genes that are implicated in the Hcy pathway were already studied in the context of MTX long-term neurotoxicity and has been found to affect neurocognitive function in childhood ALL survivors [72, 73].

*CACNB2 rs58225473* variant was associated with the neurocognitive deficit as defined by digit span test, which measures working memory. Similar risk values, although not significant, were noted in the SJLIFE cohort in all participants and in the group of survivors who received chemotherapy only. The *CACNB2* gene encodes an auxiliary voltage-dependent subunit of L-type calcium-channel that is mainly expressed in brain and heart tissue. Voltage-dependent calcium channels are crucial for neuronal differentiation and maturation; they induce large number of intracellular events such as neurotransmitter release, neuronal excitability, synaptic plasticity and gene regulation [74]. Calcium influx mediated by those channels has both spatial and temporal components and encodes important signaling information [75]. Moreover, in a recent GWAS study *CACNB2* was identified among four significant risk loci underlying genetic effects shared between five major psychiatric disorders that included schizophrenia, autism spectrum disorder, attention deficit-hyperactivity disorder, bipolar and major depressive disorders [76]. Additionally, the rare variants in this gene were found in affected members of families with autism spectrum disease [77]. Given important role that *CACNB2* can play, it is not surprising that it was studied as a possible pharmacological target in treatment of mental disorders [78]. The *rs58225473* is c.1803T>G substitution (NM_201590.2) leading to Asp601Glu replacement, which is predicted to affect protein function and possibly calcium channel function. Common variants in several other genes influenced neurocognitive decline in PETALE cohort. *PPARA* gene belongs to PPARs receptor family of ligand-activated transcription factors involved in the regulation of inflammation [79]. Effects of glucocorticoids can be reinforced by PPAR ligands [80]. The enhanced heterodimer formations of PPARA could be associated with increased expression of brain and glial cell-derived neurotrophic factors [81]. The *rs1800206* variant in *PPARA* gene was associated with lower verbal fluency score in females and survivors assigned to SR group or chemotherapy only. Similar association was noted for *ABCC3 rs12604031*. *ABCC3* is a member of the superfamily of ATP-binding cassette (ABC) transporters, and the bioavailability of MTX may be affected by this transporter [82].

*CALML5* gene is related to the calmodulin family of calcium binding proteins highly implicated in CNS function [83]. Its protective role and implication in inhibition of neuronal death was described in Alzheimer disease [84, 85]. We observed the significant association of variant *rs10904516* and deficit in verbal fluency score in all survivors with the stronger effect seen in the HR group. Similar association mostly confined to HR group was noted between lower score on trial making test and *rs2907323* in the *PCDHB10* gene. *PCDH* (protocadherin) genes, are expressed in the central and peripheral nervous systems and are required for their normal development. They mediate a variety of processes, including neuronal survival, morphogenesis and connectivity, synaptic maintenance, and spatial patterning of axons and dendrites [86]. The variants in *PCDH* genes have been reported to be associated with dyslexia and bipolar disorder [87, 88].

The neurotoxic effects of treatments in childhood ALL have been the subject of multiple investigations. These effects consist of central neurotoxicity clearly noticeable by encephalopathy and/or neurodevelopmental cognitive deficits [89–91], particularly in survivors exposed to a highly intensified treatment protocols with CNS-directed chemotherapy, even in the absence of CRT [4, 19, 92, 93]. Cognitive impairment and information processing have been associated with intensity and duration of CS treatment [94, 95]. Female survivors were reported to have more severe short-term memory impairment [59] and lower scores on attentional indices, cognitive flexibility [96, 97] and visuomotor control [4]. Female childhood ALL survivors are more likely to present cerebral white matter damage [98] that may affect cognitive functioning. Congruent with these previous observations, several associations detected in the present study were modulated by sex and treatment intensity (reflected by presence or not of CRT or risk groups).

### Anxiety and depression

We studied dimensions of internalized symptoms which are frequent in normative populations, namely anxiety and depression [99], highlighting impairment in mental quality of life of childhood ALL survivors [21, 22, 100]. Although anxiety and depression measures are not equivalent to clinical diagnosis derived from the gold standard systematic interview [101], moderate-severe levels are generally interpreted as a risk for having clinically relevant anxiety or depression.

The *rs11556218* in the *EPHA5* gene was associated with higher risk of both anxiety and depression that was further potentiated in female patients. The *EPHA5* gene codes for a brain-specific kinase that is selectively expressed in a subset of serotonin neurons during embryonic and postnatal development [102]. Receptors in the EPH subfamily modify the strength of existing synapses in the adult brain [103]. Divergent vulnerabilities between females and males could be explained by gender differences in brain maturation [104, 105], which might make females more vulnerable to the neurotoxic effects of chemotherapy. Other assumptions, such as endocrine factors, have also been hypothesized to explain sex differences in the susceptibility [59].

Moreover, we identified the association between depression and the presence of variant *rs4149056* in *SLCO1B1* gene. This association was detected in the group of survivors that received chemotherapy without CRT. *SLCO1B1* gene encodes a liver-specific member of the organic anion transporter family involved in hepatic uptake of MTX. This association deserved further attention given that the same variant was detected through genome wide association studies to contribute to inter-individual variability in the clearance of high-dose MTX [106]. It was subsequently replicated in independent cohorts and shown also as a predictor of short-term toxicity following MTX treatment [106–111]. MTX treatment has been associated with adverse emotional or behavioral outcomes [20], thus these results could justify further studies of *SLCO1B1* gene in related contexts.

### Rare variants’ analysis

The association between deficits in the trail making test score was identified in relation to rare variants enrichment in *HSPA4*, *SLCO2B1* and *GSTT1* genes, with a very strong individual contribution of *rs61745470* in *HSPA4* gene. This variant was recently associated with familial genetic risk for suicide (as well as with risk for psychiatric or substance abuse conditions) [112]. The *SLCO2B1* and *GSTT1* genes are highly implicated in physiological and pharmacological distribution of drugs and endogenous molecules. The *SLCO2B1*, a member of the organic anion transporting polypeptide (OATP) family, is involved in steroid hormone uptake and transport of steroid conjugates [113, 114]. *GSTT1* was recently associated with higher risk for early onset of severe mental and bipolar disorders [115]. We also evaluated the association of deletion polymorphisms of *GSTT1*[116] (found with the frequency of 23.7% in discovery cohort) with the deficits in the trail making test score. There was no association of *GSTT1* null genotype with the deficits in the trail making test score.

The impact of here identified rare functional variants requires further investigation.

## Concluding remarks

Our study has certain limitations. Its limited sample size may affect the accuracy of the results, particularly in the context of the stratified analysis. Other unmeasured factors in this study, for example, inflammation and oxidative stress, could modulate or potentiate association with genetic factors. The candidate gene approach may have missed genetic markers potentially involved in neurocognitive decline and mood disturbances that could have been detected through unbiased approaches. Among associations detected in the PETALE cohort only two showed a similar trend in SJLIFE cohort. Despite matching both outcomes and patients’ characteristics between the two cohorts, it is possible that small sample size, differences in treatment protocols or time of ALL diagnosis [117–120] contributed to the observed discrepancies. Likewise, stratification by risk group designation was not available for the SJLIFE cohort, precluding replication of the risk-based stratified analyses. Although the analyses in PETALE cohort were corrected for multiple testing, and confounding was reduced due to homogeneous population and uniform treatment, we cannot exclude that some of the associations have been obtained by chance.

In conclusion, using a comprehensive candidate gene approach and whole exome sequencing data we identified a panel of functionally predicted genetic variants significantly associated with neurocognitive deficits, anxiety and depression in childhood ALL survivors. Additional exome wide analysis might lead to the discovery of novel genes and genetic variants associated with neurocognitive LAEs as well as with the mood disorders.

While we acknowledge that the identified germline variants still need to be evaluated and validated through replication and functional studies, the current findings can help further understanding of the influence of genetic component on long-term complications related to cancer therapy.

## Supporting information

S1 Fig Principal component analysis (PCA). PCA analysis comparing sequencing data of 400 leukemia patients (including PETALE cohort) from Sainte-Justine University Health Center (SJUHC) to the HapMap genotype reference data (release 23) for Europeans (EUR), East Asians (EAS) and Africans (AFR).
PC1, Principal Component 1; PC2, Principal Component 2.
(TIF)

S1 Table Genotyping: Identity of polymorphisms, details of PCR and ASO hybridization. R, reverse, F, forward. The base substitution that distinguishes the two variants of each polymorphism is given in bold for ASO probes. dbSNP number is provided. Ancestral allele is given in bold and minor allele is underlined. The polymorphisms are presented as a change from ancestral to derived allele, unless ancestral allele is not known, when the change is given from major to minor allele. SNPs in coding region leading or not to amino-acid substitutions are indicated.
(DOCX)

S2 Table Significant results of association study of common variants from the candidate genes of relevance for nervous system function, PETALE cohort, WES data (n = 191). ^**a**^Association test based on comparing allele frequencies between cases and controls. All associations have FDR-BH (Benjamini-Hochberg false discovery rate) lower than 5%. All also have p value lower than 0.001, which is Bonferroni cut-off value for the number of variants tested in nervous system function pathway.
^**b**^Stratified analyses according to sex and treatment intensity (standard vs high risk); chemotherapy only vs chemotherapy and cranial radiation therapy (CRT).
Ref: reference allele; Var: variant allele; MAF: minor allele frequency, *PCDHB10*, protocadherin beta 10, *CALML5*: Calmodulin Like 5, *CACNB2*: Calcium Voltage-Gated Channel Auxiliary Subunit Beta2, *EPHA5*: EPH Receptor A5, Brain-Specific Kinase.
(DOCX)

S3 Table Results of association study of common variants from methotrexate and corticosteroids pathways, PETALE cohort, WES data (n = 191). ^**a**^Association test based on comparing allele frequencies between cases and controls. All associations have FDR-BH (Benjamini-Hochberg false discovery rate) lower than 5%.
^**b**^Stratified analyses according to sex and treatment intensity (standard vs high risk); chemotherapy only vs chemotherapy and cranial radiation therapy (CRT).
^**c**^ SNPs or associations that did not qualify for genotyping with p value higher than 0.0019 (Bonferroni cut-off value for the number of genes tested in MTX/CS pathway).
Ref: reference allele; Var: variant allele; MAF: minor allele frequency; *MTR*: 5-Methyltetrahydrofolate-Homocysteine Methyltransferase, *PPARA*: Peroxisome Proliferator Activated Receptor Alpha, *ABCC3*: ATP Binding Cassette Subfamily C Member 3, *SHMT1*: Serine Hydroxymethyltransferase 1, *ADORA3*: Adenosine Receptor A3, *SLCO1B1*: Solute Carrier Organic Anion Transporter.
(DOCX)

S4 Table The combined cohort represents the pooled samples from the discovery PETALE cohort and replication SJLIFE cohort (N = 781). Combined cohort analysis was performed for the variants in *CACNB2* and *MTR* genes. *Participants with and without indicated complications are defined as cases and controls, respectively.
**P values are calculated by Chi-square. The most representative genetic model used is indicated (d: Dominant, r: Recessive).
***Chemotherapy without cranial radiation therapy.
(DOCX)

## Abbreviations

ALL: Acute lymphoblastic leukemia
PETALE: Prévenir les Effets tardifs des Traitements de la leucémie Aiguë Lymphoblastique chez l’Enfant
SJUHC: Sainte-Justine University Health Center
LAEs: Late adverse effects
WES: Whole exome sequencing
DFCI: Dana-Faber Cancer Institute
CCSS: Childhood Cancer Survivors Study
SJLIFE: St-Jude Lifetime cohort
CRT: Cranial radiation therapy
D-KEFS: Delis-Kaplan Executive Function System
WAIS-IV: Wechsler Adult Intelligence Scale-Fourth Edition
BYI: Beck Youth Inventory
BSI-18: Brief Symptom Inventory-18
MAF: Minor allele frequency
MTX: methotrexate
CS: corticosteroids
SKAT-O test: Optimal Sequence Kernel Association Test
FDR: False discovery rate
SNP: Single nucleotide polymorphism
SR: standard risk
HR: high risk
OR: odds ratio
CI: confidence interval
CNS: central nervous system
OATP: organic anion transporting polypeptide
