# Supplementary figures and images for: Influence of genetic factors on long-term treatment related neurocognitive complications, and on anxiety and depression in survivors of childhood acute lymphoblastic leukemia: The Petale study
Source: PLoS One. 2019 Jun 10;14(6):e0217314. doi: 10.1371/journal.pone.0217314 (PMC6557490; doi:10.1371/journal.pone.0217314)

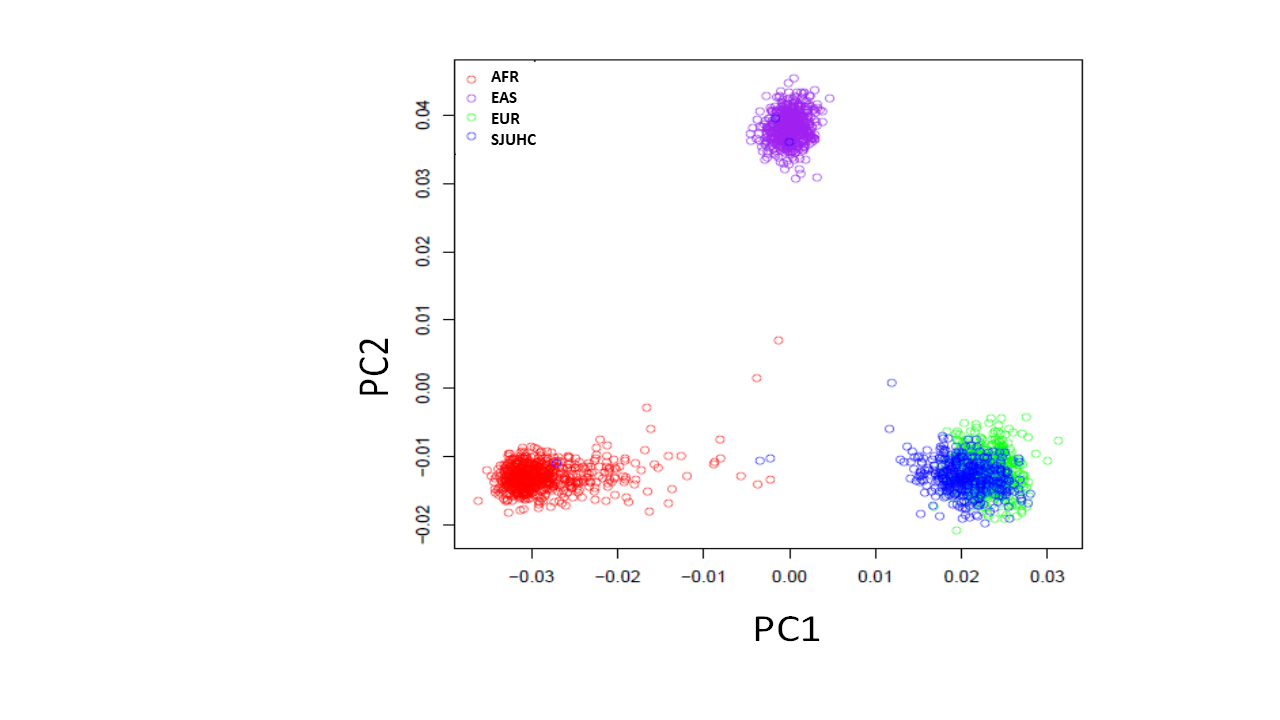

Supplement: S1 Fig — PCA analysis comparing sequencing data of 400 leukemia patients (including PETALE cohort) from Sainte-Justine University Health Center (SJUHC) to the HapMap genotype reference data (release 23) for Europeans (EUR), East Asians (EAS) and Africans (AFR). PC1, Principal Component 1; PC2, Principal Component 2. (TIF) [file pone.0217314.s001.tif]
