# Supplementary material for: Influence of genetic factors on long-term treatment related neurocognitive complications, and on anxiety and depression in survivors of childhood acute lymphoblastic leukemia: The Petale study
Source: PLoS One. 2019 Jun 10;14(6):e0217314. doi: 10.1371/journal.pone.0217314 (PMC6557490; doi:10.1371/journal.pone.0217314)
Supplement: S1 Table — R, reverse, F, forward. The base substitution that distinguishes the two variants of each polymorphism is given in bold for ASO probes. dbSNP number is provided. Ancestral allele is given in bold and minor allele is underlined. The polymorphisms are presented as a change from ancestral to derived allele, unless ancestral allele is not known, when the change is given from major to minor allele. SNPs in coding region leading or not to amino-acid substitutions are indicated. (DOCX) [file pone.0217314.s002.docx]

**S2 Table. Genotyping: Identity of polymorphisms, details of PCR and ASO hybridization.**

| **Polymorphisms** | | | | **PCR** | **ASO** | **ASO Method** |
| --- | --- | --- | --- | --- | --- | --- |
| **gene** | **dbSNP** | **position** | **variation** | **primers** | **probes** |  |
| **ABCC3** | rs12604031 | Intron | **A**/G | F: TGGGTGAGTCGGCTCCAT  R: AGCAGGTGCTCTGGATGC | CAGCCGC**G**GGTTC  CAGCCGC**A**GGTTC | ASO |
| **CACNB2** | rs58225473 | Exon | **T**/G(Asp600Glu) | Génome Québec |  | Sequenom |
| **CALML5** | rs10904516 | Exon | **T**/C(Lys74Arg) | F:CAGGCCGGCCCTGGCGTTC**T**  F:CAGGCCGGCCCTGGCGTTC**C** |  | Allele-specific PCR |
|  |  |  |  | R:TGGAAACGGCACCATCAATG |  |  |
| **EPHA5** | rs33932471 | Exon | **T**/G(Asn81Thr) | Génome Québec |  | Sequenom |
| **GSTT1** |  |  | **GSTT1/ GSTT1 null** | F:TTCCTTACTGGTCCTCACATCTC  R:TCACCGGATCATGGCCAGCA |  | PCR |
| **MTR** | rs1805087 | Exon | **A**/G(Asp919Gly) | Génome Québec |  | Sequenom |
| **PCDHB10** | rs2907323 | Exon | **C**/G (Thr213Arg)) | F:AAACTGTGGGGCATTGTCAT  R:ATTAGTGGCGGTGATGAAGG | CAGCGCT**G**TGAGGG  CAGCGCT**C**TGAGGG | ASO |
| **PPARA** | rs1800206 | Exon | **C**/G(Leu162Val) | Génome Québec |  | Sequenom |
| **SLCO1B1** | rs4149056 | Exon | **T**/C(Val174Ala) | Génome Québec |  | Sequenom |
|  |  |  |  |  |  |  |

R, reverse, F, forward. The base substitution that distinguishes the two variants of each polymorphism is given in bold for ASO probes. dbSNP number is provided. Ancestral allele is given in bold and minor allele is underlined. The polymorphisms are presented as a change from ancestral to derived allele, unless ancestral allele is not known, when the change is given from major to minor allele. SNPs in coding region leading or not to amino-acid substitutions are indicated.
