# Supplementary material for: Influence of genetic factors on long-term treatment related neurocognitive complications, and on anxiety and depression in survivors of childhood acute lymphoblastic leukemia: The Petale study
Source: PLoS One. 2019 Jun 10;14(6):e0217314. doi: 10.1371/journal.pone.0217314 (PMC6557490; doi:10.1371/journal.pone.0217314)
Supplement: S2 Table — aAssociation test based on comparing allele frequencies between cases and controls. All associations have FDR-BH (Benjamini-Hochberg false discovery rate) lower than 5%. All also have p value lower than 0.001, which is Bonferroni cut-off value for the number of variants tested in nervous system function pathway. bStratified analyses according to sex and treatment intensity (standard vs high risk); chemotherapy only vs chemotherapy and cranial radiation therapy (CRT). Ref: reference allele; Var: variant allele; MAF: minor allele frequency, PCDHB10, protocadherin beta 10, CALML5: Calmodulin Like 5, CACNB2: Calcium Voltage-Gated Channel Auxiliary Subunit Beta2, EPHA5: EPH Receptor A5, Brain-Specific Kinase. (DOCX) [file pone.0217314.s003.docx]

**S2 Table.** **Significant results of association study of common variants from the candidate genes of relevance for nervous system function, PETALE cohort, WES data (n=191).**

| Outcome | Gene | SNP | Ref allele | Var allele | MAF | | P value ^a^ | | Allelic ratio frequencies | |
| --- | --- | --- | --- | --- | --- | --- | --- | --- | --- | --- |
|  |  |  |  |  |  |  |  |  | **AFF** | **UNAFF** |
| Trial making test | **High risk^b^, N=108** | | | | | | | | | |
|  | *PCDHB10* | rs2907323 | C | G | | 0.13 | 0.0006 | 9/17 | | 19/167 |
| Verbal fluency | **All cohort, N=191** | | | | | | | | | |
|  | *CALML5* | rs10904516 | T | C | 0.24 | | 0.0002 | | 29/43 | 56/230 |
|  | **High risk^b^, N=108** | | | | | | | | | |
|  | *CALML5* | rs10904516 | T | C | 0.24 | | 0.0002 | | 20/24 | 28/128 |
| Digit span | **Males, N=87** | | | | | | | | | |
|  | *CALML5* | rs10904516 | T | C | 0.22 | | 0.0003 | | 16/20 | 21/111 |
|  | **Chemotherapy only, N=79** | | | | | | | | | |
|  | *CACNB2* | rs58225473 | T | G | 0.17 | | 0.0004 | | 9/11 | 18/120 |
| Moderate-severe anxiety | **Females, N=104** | | | | | | | | | |
|  | *EPHA5* | rs33932471 | T | G | 0.08 | | 0.00003 | | 6/12 | 9/163 |
| Moderate-severe depression | **All cohort, N=191** | | | | | | | | | |
|  | *EPHA5* | rs33932471 | T | G | 0.06 | | 0.0004 | | 8/36 | 13/283 |
|  | **Females, N=104** | | | | | | | | | |
|  | *EPHA5* | rs33932471 | T | G | 0.08 | | 0.0003 | | 7/21 | 8/154 |

**^a^**Association test based on comparing allele frequencies between cases and controls. All associations have FDR-BH (Benjamini-Hochberg false discovery rate) lower than 5%. All also have p value lower than 0.001, which is Bonferroni cut-off value for the number of variants tested in nervous system function pathway.

**^b^**Stratified analyses according to sex and treatment intensity (standard vs high risk); chemotherapy only vs chemotherapy and cranial radiation therapy (CRT).

Ref: reference allele; Var: variant allele; MAF: minor allele frequency, *PCDHB10:* protocadherin beta 10, *CALML5*: Calmodulin Like 5, *CACNB2*: Calcium Voltage-Gated Channel Auxiliary Subunit Beta2, *EPHA5*: EPH Receptor A5, Brain-Specific Kinase.
