# Supplementary material for: Influence of genetic factors on long-term treatment related neurocognitive complications, and on anxiety and depression in survivors of childhood acute lymphoblastic leukemia: The Petale study
Source: PLoS One. 2019 Jun 10;14(6):e0217314. doi: 10.1371/journal.pone.0217314 (PMC6557490; doi:10.1371/journal.pone.0217314)
Supplement: S3 Table — aAssociation test based on comparing allele frequencies between cases and controls. All associations have FDR-BH (Benjamini-Hochberg false discovery rate) lower than 5%. bStratified analyses according to sex and treatment intensity (standard vs high risk); chemotherapy only vs chemotherapy and cranial radiation therapy (CRT). c SNPs or associations that did not qualify for genotyping with p value higher than 0.0019 (Bonferroni cut-off value for the number of genes tested in MTX/CS pathway). Ref: reference allele; Var: variant allele; MAF: minor allele frequency; MTR: 5-Methyltetrahydrofolate-Homocysteine Methyltransferase, PPARA: Peroxisome Proliferator Activated Receptor Alpha, ABCC3: ATP Binding Cassette Subfamily C Member 3, SHMT1: Serine Hydroxymethyltransferase 1, ADORA3: Adenosine Receptor A3, SLCO1B1: Solute Carrier Organic Anion Transporter. (DOCX) [file pone.0217314.s004.docx]

**S3 Table. Results of association study of common variants from methotrexate and corticosteroids pathways, PETALE cohort, WES data (n=191).**

| Outcome | Gene | SNP | Ref allele | Var allele | MAF | P value ^a^ | Allelic ratio frequencies | |
| --- | --- | --- | --- | --- | --- | --- | --- | --- |
|  |  |  |  |  |  |  | **AFF** | **UNAFF** |
| Verbal fluency | **Males, N=87 ^b^** | | | | | | | |
|  | *MTR* | rs1805087 | A | G | 0.19 | 0.0006 | 14/22 | 19/119 |
|  | **Standard risk, N=83** | | | | | | | |
|  | *PPARA* | rs1800206 | C | G | 0.07 | 0.0001 | 7/21 | 5/133 |
|  | **Females, N=104** | | | | | | | |
|  | *PPARA* | rs1800206 | C | G | 0.07 | 0.0003 | 6/30 | 4/168 |
|  | **High risk, N=108** | | | | | | | |
|  | *ABCC3* | rs12604031 | G | A | 0.44 | 0.0006 | 25/11 | 50/84 |
|  | **Chemotherapy only, N=79** | | | | | | | |
|  | *PPARA* | rs1800206 | C | G | 0.06 | 0.00005 | 6/18 | 4/130 |
|  | **Chemotherapy and CRT, N=112** | | | | | | | |
|  | *SHMT1***^c^** | rs1979277 | G | A | 0.27 | 0.002 | 21/25 | 35/123 |
|  | *ABCC3* | rs12604031 | G | A | 0.47 | 0.003 | 27/13 | 55/81 |
| Moderate-severe depression | **Standard risk, N=83** | | | | | | | |
|  | *ADORA3***^c^** | rs35511654 | T | G | 0.12 | 0.002 | 6/12 | 10/112 |
|  | **Chemotherapy only, N=79** | | | | | | | |
|  | *SLCO1B1* | rs4149056 | T | C | 0.15 | 0.0001 | 8/10 | 12/108 |
|  | *ADORA3***^c^** | rs35511654 | T | G | 0.10 | 0.004 | 5/13 | 8/112 |
|  | *ABCC3^c^* | rs11568591 | G | A | 0.07 | 0.004 | 4/14 | 5/115 |

**^a^**Association test based on comparing allele frequencies between cases and controls. All associations have FDR-BH (Benjamini-Hochberg false discovery rate) lower than 5%.

**^b^**Stratified analyses according to sex and treatment intensity (standard vs high risk); chemotherapy only vs chemotherapy and cranial radiation therapy (CRT).

**^c^** SNPs or associations that did not qualify for genotyping with p value higher than 0.0019 (Bonferroni cut-off value for the number of genes tested in MTX/CS pathway).

Ref: reference allele; Var: variant allele; MAF: minor allele frequency; *MTR*: 5-Methyltetrahydrofolate-Homocysteine Methyltransferase, *PPARA*: Peroxisome Proliferator Activated Receptor Alpha, *ABCC3*: ATP Binding Cassette Subfamily C Member 3, *SHMT1*: Serine Hydroxymethyltransferase 1, *ADORA3*: Adenosine Receptor A3, *SLCO1B1*: Solute Carrier Organic Anion Transporter.
