# Supplementary material for: Influence of genetic factors on long-term treatment related neurocognitive complications, and on anxiety and depression in survivors of childhood acute lymphoblastic leukemia: The Petale study
Source: PLoS One. 2019 Jun 10;14(6):e0217314. doi: 10.1371/journal.pone.0217314 (PMC6557490; doi:10.1371/journal.pone.0217314)
Supplement: S4 Table — *Participants with and without indicated complications are defined as cases and controls, respectively. **P values are calculated by Chi-square. The most representative genetic model used is indicated (d: Dominant, r: Recessive). ***Chemotherapy without cranial radiation therapy. (DOCX) [file pone.0217314.s005.docx]

**S4 Table.** **The combined cohort represents the pooled samples from the discovery PETALE cohort and replication SJLIFE cohort (N=781). Combined cohort analysis was performed for the variants in *CACNB2* and *MTR* genes.**

| **Outcome** | **Genotype** | **Case* N (%)** | **Control* N (%)** | **Model** | **Case* N (%)** | **Control* N (%)** | **P**** | **OR (95%-CI)** |
| --- | --- | --- | --- | --- | --- | --- | --- | --- |
| **Digit span** | ***CACNB2 rs58225473*** | | | | | | | |
|  | **All patients** | | | | | | | |
|  | TT | 48 (64.0) | 407 (69.8) | TT+TG | 70 (93.3) | 572 (98.11) | 0.01^r^ | 3.7 (1.25-11) |
|  | TG | 22 (29.3) | 165 (28.3) |  |  |  |  |  |
|  | GG | 5 (6.7) | 11 (1.9) | GG | 5 (6.7) | 11 (1.89) |  |  |
|  | **Chemotherapy only***** | | | | | | | |
|  | TT | 20 (58.8) | 175 (68.4) | TT+TG | 29 (85.3) | 250 (97.7) | 0.0004^r^ | 7.2 (2.1-25) |
|  | TG | 9 (26.5) | 75 (29.3) |  |  |  |  |  |
|  | GG | 5 (14.7) | 6 (2.3) | GG | 5 (14.7) | 6 (2.3) |  |  |
| **Verbal fluency** | ***MTR rs1805087*** | | | | | | | |
|  | **All patients** | | | | | | | |
|  | AA | 67(59.8) | 443 (69.6) | AA | 67 (59.8) | 443 (69.6) | 0.04^d^ | 1.5 (1-2.3) |
|  | AG | 37 (33.0) | 179 (28.1) | AG+GG | 45 (40.2) | 194 (30.5) |  |  |
|  | GG | 8 (7.2) | 15 (2.4) |  |  |  |  |  |
|  |  | | | | | | | |
|  | AA | 35 (57.4) | 221 (70.6) | AA | 35 (57.4) | 221 (70.6) | 0.04^d^ | 1.8 (1-3.1) |
|  | AG | 20 (32.8) | 85 (27.2) | AG+GG | 26 (42.6) | 92 (29.4) |  |  |
|  | GG | 6 (9.8) | 7 (2.2) |  |  |  |  |  |

*Participants with and without indicated complications are defined as cases and controls, respectively

**P values are calculated by Chi-square. The most representative genetic model used is indicated (d: Dominant, r: Recessive).

***Chemotherapy without cranial radiation therapy.
